# Liver Phantoms Cast in 3D-Printed Mold for Image-Guided Procedures

**DOI:** 10.3390/diagnostics14141521

**Published:** 2024-07-15

**Authors:** Radu Claudiu Elisei, Florin Graur, Andreas Melzer, Sever Calin Moldovan, Calin Tiu, Calin Popa, Emil Mois, Doina Pisla, Calin Vaida, Horia Ștefănescu, Adrian Coțe, Nadim Al-Hajjar

**Affiliations:** 1 Department of Surgery, University of Medicine and Pharmacy “Iuliu Hatieganu”, 400012 Cluj-Napoca, Romania; radu_elisei@yahoo.com (R.C.E.); calinp2003@yahoo.com (C.P.); drmoisemil@gmail.com (E.M.); horia.stefanescu@irgh.ro (H.Ș.); na_hajjar@yahoo.com (N.A.-H.); 2 Emergency County Hospital, 420016 Bistrita, Romania; severcalinmoldovan@gmail.com; 3 Regional Institute of Gastroenterology and Hepathology “Dr. Octavian Fodor”, 400394 Cluj-Napoca, Romania; 4 CESTER Department, Faculty of Industrial Engineering, Robotics and Production Management, Technical University of Cluj-Napoca, 400114 Cluj-Napoca, Romania; doina.pisla@mep.utcluj.ro (D.P.); calin.vaida@mep.utcluj.ro (C.V.); 5 ICCAS Institute of Computer Assisted Surgery, University Leipzig, 04103 Leipzig, Germany; a.melzer@dundee.ac.uk; 6 IMSAT Institute for Medical Science and Technology, University Dundee, Dundee DD1 9SY, UK; 7 Municipal Hospital, 105600 Campina, Romania; tiucalin@yahoo.com; 8 Emergency County Hospital, 410159 Oradea, Romania; adrian.cote@gmail.com

**Keywords:** gelatin-based phantom, liver mold, liver surgery, liver biopsy, training, ultrasound guided, image guided, diagnostic training

## Abstract

Introduction: Image-guided invasive procedures on the liver require a steep learning curve to acquire the necessary skills. The best and safest way to achieve these skills is through hands-on courses that include simulations and phantoms of different complications, without any risks for patients. There are many liver phantoms on the market made of various materials; however, there are few multimodal liver phantoms, and only two are cast in a 3D-printed mold. Methods: We created a virtual liver and 3D-printed mold by segmenting a CT scan. The InVesalius and Autodesk Fusion 360 software packages were used for segmentation and 3D modeling. Using this modular mold, we cast and tested silicone- and gelatin-based liver phantoms with tumor and vascular formations inside. We tested the gelatin liver phantoms for several procedures, including ultrasound diagnosis, elastography, fibroscan, ultrasound-guided biopsy, ultrasound-guided drainage, ultrasound-guided radio-frequency ablation, CT scan diagnosis, CT–ultrasound fusion, CT-guided biopsy, and MRI diagnosis. The phantoms were also used in hands-on ultrasound courses at four international congresses. Results: We evaluated the feedback of 33 doctors regarding their experiences in using and learning on liver phantoms to validate our model for training in ultrasound procedures. Conclusions: We validated our liver phantom solution, demonstrating its positive impact on the education of young doctors who can safely learn new procedures thus improving the outcomes of patients with different liver pathologies.

## 1. Introduction

Invasive procedures performed on the liver require a steep learning curve related to acquiring the necessary skills to perform both open and laparoscopic surgical maneuvers, during intraoperative liver ultrasound (US) and interventional ultrasound (percutaneous, intraoperative open, or laparoscopic procedures).

There is no standardized minimum number of procedures required for a physician to be considered competent in performing US-guided liver punctures (biopsies). However, one of the criteria to obtain accreditation for advanced training in hepatology in the USA (Gastrointestinal Core Curriculum on the American Gastroenterological Association) is to have performed 40 US-guided liver biopsies under supervision [1]. In Europe, there are currently no mandatory criteria for obtaining accreditation for this type of procedure.

The role of hands-on workshops is to develop the skills needed to perform such procedures, but they are rare and expensive, with fees ranging from a few hundreds to a few thousand Euros, depending on their complexity, the number of participants, and the percentage of time represented by the hands-on section of the course.

A suitable training program is essential, since it leads to a faster learning curve and more effective acquisition of the skills needed to perform such procedures. In addition, allowing surgeons to learn both the basics and very specific procedures on phantoms significantly improves the safety of patients and, at the same time, has the potential to increase the number of competent surgeons in this field. Given the continuous development of medical specialties, conventional ultrasounds, especially intraoperative ultrasounds, are an important aspect of the training of a surgeon. Thus, the training for interventional ultrasounds should address the needs of the surgeons.

Liver phantoms with tumors and vascular structures [2] are available; some have a realistic appearance [2,3] or blood flow functionality [4]. Some liver phantoms offer the possibility of training for US-guided punctures and ultrasound navigation in a transverse abdominal segment that contains both liver and vascular structures and can be used for US, computer tomography (CT), and magnetic resonance imaging (MRI), e.g., the Triple Modality 3D Abdominal Phantom Model 0557A (CIRS Inc., Norfolk, VA, USA) [5], available for USD 2897 [6]. Another liver phantom that includes the organs of the upper abdomen is dedicated to open intraoperative or laparoscopic US, the Abdominal Intraoperative and Laparoscopic Ultrasound Phantom–IOUSFAN, produced by Kyoto Kagaku Co., Ltd. (Kyoto, Japan) [7] and available at the price of USD 8700 [8].

Multimodal liver phantoms are rare [2,3,9,10] and only one provides blood flow functionality [4]. The majority of liver phantoms are designed for imaging, including CT [11,12,13,14], MRI [15,16], and US [17,18,19]. Only a few of them reproduce the real external appearance of the liver [4,17,19,20], while a few have vascular structures inside [4,16,17,19], and only two of them are cast in a 3D-printed (3DP) mold [19,20], which is the easiest and most affordable method to create liver phantoms with a real liver appearance.

Compared to all these liver phantoms, the model created by our team is an affordable multimodal liver phantom with an anatomical external appearance and tumors and vascular structures inside. It is easy to produce and customize, and is designed for training in US-, CT-, and MRI-guided procedures, without providing blood flow functionality or an artificial abdominal wall. The majority of the liver phantoms presented above are experimental models, except the Triple Modality 3D Abdominal Phantom Model 0557A [6] and the Abdominal Intraoperative and Laparoscopic Ultrasound Phantom–IOUSFAN [7].

Due to the scarcity of accessible anatomical liver phantoms for training in image-guided procedures, we have developed and validated a novel liver phantom cast in a 3D-printed mold. This phantom has been used in multiple hands-on training courses for over three years.

Regarding soft liver phantoms, several substances have been used in the literature to mimic liver parenchyma. A review of these was prepared by Culjat M.O. et al. [21] in 2010, in which multiple such solutions were described as follows: gelatin-based, agarose-based, magnesium silicate-based, oil gel-based, open-cell foam-based, polyacrylamidegel-based, polyurethane gel-based, and organic-based solutions [21]. Scientific technical gelatin (ballistic gel) can also be used [22]. While gelatin-based phantoms are sensitive to the environmental temperature, melting at high temperatures (over 35 °C), bi-component silicone phantoms cure at room temperature.

A recipe described by Bude and Alder for a gelatin-based clear and opaque liver phantom uses 20g of dehydrated gelatin for every 20 mL water and psyllium hydrophilic mucilloid fiber [23].

The novelty of this work is the development of a simple-to-cast and personalized liver phantom that can be used for the training of young surgeons for any image-guided procedure. Based on its multi-use cast, it enables the production of any specific internal anatomy in a time frame of less than 24 h, and with easy-to-find products, resulting in an efficient, adaptable training tool for a large number of minimally invasive liver procedures.

The goal of this work was the creation of an easily available, feasible, easy-to-reproduce, easy-to-use, inexpensive, and effective training alternative for acquiring skills in image-guided procedures. To achieve this, we tried to produce a 3DP mold with a unit cost of below EUR 1000 to be used to cast versatile and multimodal liver phantoms with a unit cost of below EUR 100, as well as a real human liver shape and size, to be used for training in different image-guided procedures.

## 2. Materials and Methods

### 2.1. Making of the Mold

#### 2.1.1. Virtual Mold

Using InVesalius (open-source software) we started with a stereolithography (STL) file obtained from an accurate segmentation of the parenchyma of a normal liver from a 67-year-old female patient from a 64 slice CT scan DICOM file, with i.v. contrast and a slice thickness of 1.25 mm (GE Revolution EVO X-ray system GMDN code: 37618, GE Healthcare Japan Corporation, Tokyo, Japan).

The DICOM images were opened in InVesalius (Centro de Tecnologia da informação Renato Archer CTI, InVesalius 3, open-source software, Campinas, Brazil). The liver parenchyma was selected using the “region growing” button, and then the unwanted regions were removed using the “crop” or “delete” button, leaving only a 3D model of the liver.

To obtain a smooth surface of the liver, the “smooth” button from the “surface” menu was used and then the “close holes” button from the same menu was used to close the holes and pores on the surface of the 3D model, followed by the generation of the STL file.

The STL file was opened in Autodesk Fusion 360 with Netfabb (Autodesk software company, Fusion 360 2.0.19426 with Autodesk Netfabb Premium 64-Bit Edition, San Francisco, CA, USA) for 3D modeling.

**Figure 1:**
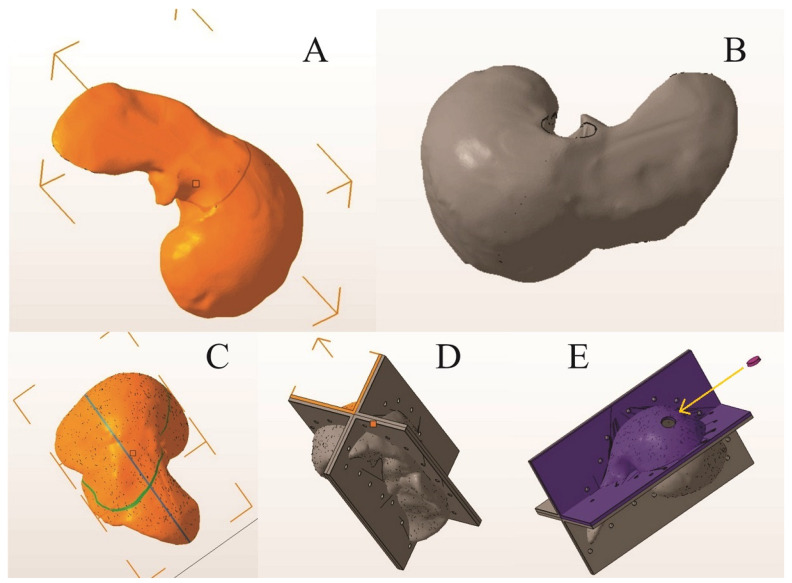
Virtual 3D reconstruction of the liver (Autodesk Fusion 360 with Netfabb) (**A**,**B**) and virtual liver mold: (**C**) section lines; (**D**) the 4 segments assembled; (**E**) casting access with the cap for the casting hole. (The arrow show the place where the cap should be placed on the casting hole).

- After opening the STL file, the 3D model of the liver was selected and, using the “automatic repair” function, possible integrity problems of the model were checked and corrected.
- Using the “cut” and “delete” functions, the unwanted parts and overlapping geometries of the model were eliminated and adjusted.
- Later, the manual repair functions were used for any remaining problems (small holes or unfavorable geometry).
- Using the “measure” function, the correct dimensions of the model were checked and the number of triangles on the surface of the model was optimized using the “reduce” function (this function reduces the size of the file and improves its performance for 3D printing).
- Using the “extrude” function, a thickness of 5 mm was given to the outer surface (Figure 1A,B), and with the “cut” function, the 3D model was sectioned into 4 segments, so that demolding could be carried out without damaging the finished product, considering the irregular shape of the human liver (Figure 1C).
- Geometries were created on the 4 segments using the same “extrude” function.
- Using the “Boolean” function, assembly/fixing holes were created on these geometries through which screws could be mounted. The dimensions of the assembled mold were a length of 300 mm, a width of 200 mm, and a height of 200 mm (Figure 1D).
- Using the “cut” function, a casting hole was created on the quarter of the mold with the highest point, specifically in the quarter that reproduces the surface of segments VII and VIII of the liver; the cut region could be used as a cap for the casting hole (Figure 1E).

Then, using the “export” function, the repaired STL file was saved.

#### 2.1.2. Physical Mold

**Figure 2:**
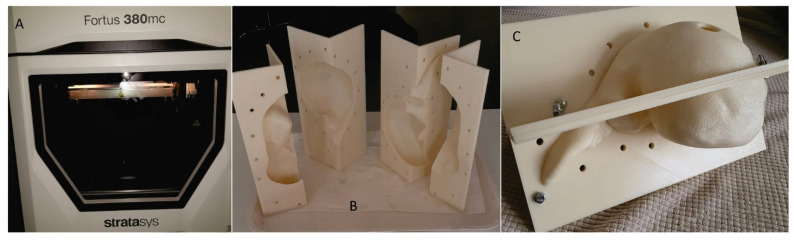
Real liver mold: (**A**) 3D printer; (**B**) the 4 segments of the mold; (**C**) liver mold assembled.

After making the 3D-printable STL files of the 4 quarters of the mold, the 3D printing was carried out using ABS (ABS-M30^TM^ Model, Stratasys 3D printing company, Minneapolis, MN, USA), with fused deposition modeling (FDM) technology, using a professional 3D printer (Stratasys Fortus 380mc, Stratasys 3D printing company, Fortus 380 mc 3D printer, Minneapolis, MN, USA) (Figure 2A). The layer thickness was 0.256 mm, with a deviation of less than 1%. By using a professional 3D printer with a large print base, very thin layers of the materials could be printed with high fidelity and minimal deviation (<1%) compared to the printable file. The mold had accurate temperature control, a heated bed, and soluble support material that could be removed without any mechanical stress on the actual part. The actual mold cavity volume was 1533.7 cm^3^. According to Andersen V et al. [24], the average liver volume of a healthy adult is 1541 cm^3^, and according to Agrawal D et al. [25], the average liver volume of an adult between the ages of 21 and 70 is 1445.2 cm^3^.

After 3DP of the 4 segments of the mold, the support material (SR-30^TM^ Soluble Support, Stratasys 3D printing company, Minneapolis, MN, USA) was dissolved using a special solution (P400SC^TM^ Waterworks^TM^ Cleaning Solution, Keteca Inc., Phoenix, AZ, USA), obtaining the 4 clean segments (Figure 2B,C). Since materials printed using the FDM method is porous, which allows liquids to infiltrate, both the inner and outer surface of the mold components were sealed using industrial acetone-based varnish for plastics. After the varnish dried, the inside of the mold was covered with a layer of wax (Kent–Impermea Grasso, neutral waterproof dubbin, based on paraffin, https://www.benvenuti.com/ro/prestige/crema-impermeabila accessed on 2 August 2021) for very easy release/removal of the liver phantom. Sealing between the mold elements was carried out using sanitary silicone (Ceresit-Sanitary Express, transparent silicone, https://www.dedeman.ro/ro/silicon-sanitar-transparent-ceresit-cs-25-interior/-exterior-280-ml/p/5001014 accessed on 2 August 2021), which was easily removed after the unmolding.

### 2.2. Making of a Liver Phantom

**Figure 3:**
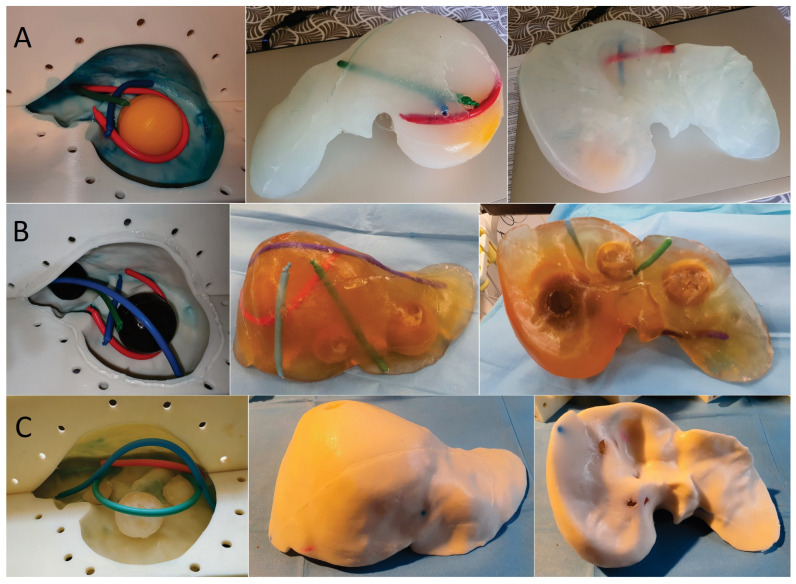
(**A**) Casting silicone-based liver phantom; (**B**) casting transparent gelatin-based liver phantom; (**C**) casting opaque gelatin-based liver phantom.

In order to prove the effectiveness of the mold two types of liver phantoms were made from different materials as a basis for the liver parenchyma and tumor formations. We made one clear and one opaque gelatin-based phantom using pork/beef dry gelatin (14g of gelatin and a similar amount of sugar for every 100 mL of water). For the opaque phantom, we used milk cream for a scattering effect. The tumor formations inside both gelatin phantoms were made from the same material as the parenchyma; for the clear phantom, we added gelatin-based tumors with contrast (milk cream), and for the opaque one, we used clear gelatin tumors (Figure 3B,C). For a scattering effect in gelatin phantoms, wheat flour, corn starch, or talcum powder can also be used. The second material we used to create a liver phantom was ZA13 bi-component Shore-13 scale silicone (Zhermack-ZA 13 MOULD WT 45, btools.ro accessed on 2 August 2021). The tumor formations in this phantom were made of the same material with a color solution for the silicone (Figure 3C). In both the gelatin and silicone phantoms, the tumor formations were cast separately, using ice cube bags for small tumors and silicone sphere ice molds for large tumors, and then incorporated into the phantom. In order to simulate the large vascular axes of the liver in both types of phantoms, we decided to use modeling balloons (Gemar latex modeling balloons, emag.ro), with a maximum diameter of 10 mm filled with red- and green-colored water/ink.

Liver phantom casting steps:

- The 3 segments of the mold were assembled, with the exception of the segment with the casting hole (Figure 3), using screws for fixing and sanitary silicone for sealing.
- The tumor formations were placed inside, on the mold’s base (Figure 3).
- Vascular formations were also placed inside, around the tumors, on the mold’s base (Figure 3).
- The base liquid of the phantom (gelatin-based liquid or bi-component silicone) was poured inside the mold to the level allowed, with only 3 of the mold segments mounted.
- The last element of the mold was assembled in the same way as the first 3, and we continued to pour the base liquid through the pouring hole up to the level of the hole.
- The bi-component silicone phantom was left to solidify in the mold at room temperature for 6 h, and the gelatin-based phantom was left in the refrigerator at 2–4 °C for gelabased phantom tin-based phantom.
- After solidification, we proceeded to demold the liver phantoms by removing the fixing screws and carefully removing the mold segments one at a time (Figure 3).
- For the silicone liver, after the ZA13 transparent bi-component silicone solution was homogenized at room temperature, it was placed in a vacuum vessel for 10 min to extract the air bubbles inside the composition before it was poured inside the mold cavity. After demolding, a silicone liver phantom with semi-transparent parenchyma was obtained, through which the tumor formations and the simulated vascular axes could be seen (Figure 3A).
- For the gelatin-based liver phantom, after homogenization at 40 °C, the gelatin solution was allowed to cool for 15 min at room temperature, since pouring at a high temperature can lead to partial or total dissolution of already solidified tumors mounted inside the mold. After the liquid temperature dropped below 20 °C, it was slowly poured into the mold cavity as described above. After demolding, a gelatin-based liver phantom with transparent parenchyma was obtained, through which the tumor formations and vascular structures could be seen (Figure 3B,C).

### 2.3. Testing of the Gelatin-Based Liver Phantom

The liver phantoms made of silicone and gelatin were examined by our team (8 surgeons (5 with ultrasound competence), 3 engineers, and 1 gastroenterologist) via palpation, US, elastography, the Fibroscan device, and US-guided puncture/biopsy. The high puncture resistance of the silicone phantom made the procedure very difficult. Due to the easy production and reduced cost of the gelatin-based phantom (15% of the silicone-based phantom), further testing was performed only with the gelatin-based phantom, including US-guided biliary drainage, US-guided radio frequency ablation (RFA) needle insertion, CT examination, MRI examination, US–CT fusion examination, laparoscopic US-guided puncture, and CT-guided puncture (Table 1). Its resistance to manipulation and multiple punctures was tested, along with its durability over time.

We decided to validate our work by using the gelatin-based liver phantoms at hands-on courses on ultrasound (with 4 modules: abdominal US, US-guided procedures, intraoperative US, and trauma US):

- World Congress for Endoscopic Surgery (WCES; Barcelona, 2021) [26]: 29 participants (13 participants attended the guided procedures and intraoperative modules);
- Congress of European Association for Endoscopic Surgery (EAES; Krakow, 2022) [27]: 22 participants (9 participants attended the guided procedures and intraoperative modules);
- Congress of the Romanian Association for Endoscopic Surgery (RAES; Timisoara, 2022) [28]: 12 participants (7 participants attended both the guided procedures and trauma US modules);
- EAES Winter Meeting (Malta, 2023) [29]: 13 participants (6 participants attended intraoperative module and 7 attended both guided procedures and trauma US modules).

All the participants who evaluated the phantoms were residents and specialist surgeons without ultrasound competence or any experience in ultrasound procedures. The participants of the guided procedures and intraoperative US modules performed training on gelatin liver phantoms. In each course, the participants used the phantoms for diagnostic US, US-guided punctures, biopsies and cyst evacuation, and US-guided biliary drainage. They also used non-anatomical gelatin-based phantoms for the same procedures.

During the hands-on courses, all the participants successfully used our liver phantoms without any difficulties in the identification of the internal structures using an US or in the guided procedures. Following the completion of the courses, we asked the participants from the “US-guided” module within the ultrasound course to complete an online Likert-scale questionnaire for the evaluation of the phantoms. In the questionnaire, there were 9 questions regarding their experience with the phantoms (Table 2) and 1 question for suggestions to improve the phantoms. We obtained 33 responses overall.

In the end, we evaluated all the answers of the participants to assess the life-sized gelatin liver phantom for training in diagnostic US and other US-guided procedures. The participants evaluated the gelatin liver phantoms only from a qualitative point of view compared to non-anatomical gelatin phantoms used for training on the same diagnostic US and US-guided procedures during hands-on courses.

The Likert scale questionnaire was as follows:

Q1: How real/natural did you think the gelatin liver phantoms used in the course were?
Q2: What do you think about the general appearance of the anatomical gelatin liver phantoms?
Q3: What do you think about the quality of the anatomical gelatin liver phantoms used in the course?
Q4: What do you think about the consistency of the anatomical gelatin liver phantoms used in the course?
Q5: How satisfied are you with regard to the durability of the anatomical gelatin liver phantoms for repeated ultrasound guided punctures?
Q6: How satisfied are you with the ultrasound images produced by the anatomical gelatin liver phantoms?
Q7: How well can you practice ultrasound-guided punctures on the anatomical gelatin liver phantoms used in the course?
Q8: How useful do you think the use of anatomical gelatin liver phantoms is for ultrasound-guided puncture skill development?
Q9: Which molds did you find more useful: the anatomical ones of the liver or the simple NON-anatomical ones?

## 3. Results

We made a 3DP mold composed of four pieces, allowing for the casting of phantoms with the actual shape and size of a human liver from gelatin- or silicone-based materials. The manufacturing time of the mold was 42 h. Liver phantoms cast in this mold was able to include simulated vascular structures and tumor formations of various sizes.

The overall cost of manufacturing the mold was about EUR 1000 (NUtechnologies Ltd., Timișoara, Romania). The cost of the gelatin-based liver phantom was between EUR 8 and 15, and that of the silicone liver phantom was between EUR 70 and 90. The mold can be used more than 100 times (tested) without any damage. The designs of the phantoms cast in this mold can vary greatly, depending on the user’s need, including vascular structures of varying sizes and tumor formations and collections of varying sizes, all of which can be used for training with US, CT, or MRI. The time required to make a phantom was about 6 h.

**Figure 4:**
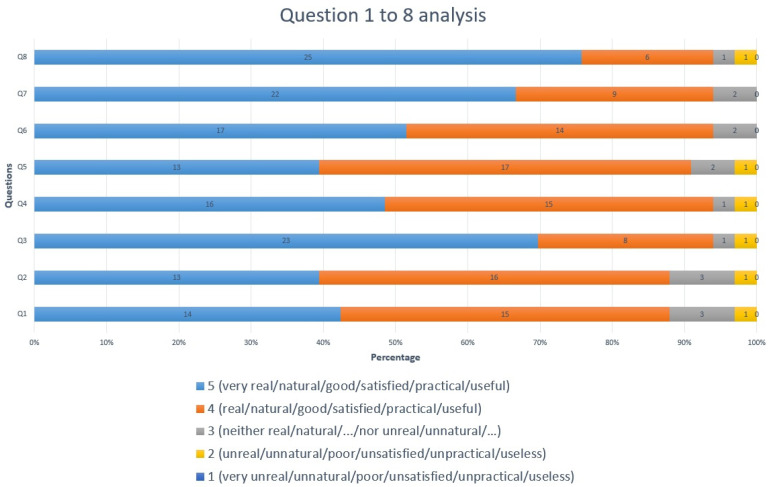
Questionnaire assessment: Analysis of questions 1 to 8.

**Figure 5:**
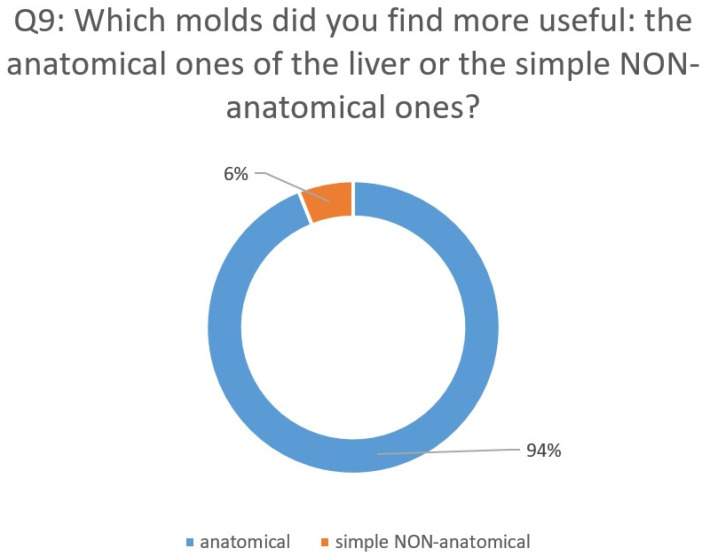
Questionnaire assessment: Analysis of question 9.

After evaluating the 33 responses to the questionnaire from the participants of the hands-on ultrasound courses listed above, we found that our gelatin-based liver phantoms were very useful and feasible to be used for the acquisition of ultrasound procedures skills. The participants answered 10 different questions regarding their experience with the gelatin liver phantoms (Figure 4 and Figure 5).

For the 10th question, when it came to suggestions regarding gelatin liver phantoms, we received only the following two answers: blood vessels and perfusion.

The participants answered with percentages of 94% to six questions, 91% to one question, and 88% to two questions that they were “very satisfied” or “satisfied” (5 or 4 points, respectively, out of a maximum of 5) with the experience they had with the gelatin liver phantoms during the EAES and RAES hands-on ultrasound courses.

## 4. Discussion

Our team has over 10 years of experience in the hands-on training of surgeons, including both residents and specialists in laparoscopic and open surgery. During the last 5 years, we have spent a lot of time developing innovative and accessible training techniques for liver tumor interventional procedures and rectal laparoscopic surgery using 3DP. As a result of this research, we managed to develop these gelatin-based and silicon-based liver models.

Imaging-guided liver procedures require a learning curve [30]. In the case of RFA for non-resectable primary or metastatic liver tumors, it has been shown that there was a learning curve of 50 cases of the procedure before a significant improvement in results was observed [31]. Additionally, according to the American Association for the Study of Liver Diseases (AASLD), the minimum number of procedures required for proficiency in percutaneous liver biopsy is 40 biopsies [1]. This liver model, with variable-sized tumors and blood vessels throughout, could significantly reduce the learning curve in terms of the number of patients.

In the literature, we identified two articles that describe the production process of a 3D-printed mold for creating liver phantoms. Witowski JS et al. [20] used an FDM desktop 3D printer to create and 3D print a mold in which the phantom of a silicone liver was cast for preoperative planning. Pacioni A et al. [19] used an FDM desktop 3D printer to create a 3DP mold with which he developed a patient-specific liver phantom dedicated to liver US training. Neither of these molds were made with a high-precision 3D printer, and the assembly of the molds suffered from problems due to the lower quality of the 3DP. In the first case [20], the liver phantom was made for preoperative planning, and in the second case [19], a multiple-use phantom was made for training. Due to the poor assembly of the mold elements, in both cases, gelatin liver phantoms cannot be made with these molds due to the very low viscosity of the gelatin solution compared to silicone when it is poured into the mold.

Most 3D printers use either FDM or PolyJet technology. On the market, there are many commercial services available that perform 3DP using a variety of grades of equipment [32,33]. The printer used by our team to print the mold was the Stratasys Fortus 380mc as it enables the correct printing of large parts due to the use of a high-quality printing filament, a closed printing chamber with real-time temperature control, and a set of the following two types of printing materials: the ABS filament for the part and a soluble material for support, which allows for much better detailing of any cavities and non-contact removal of the support, which preserves the quality of the printed surfaces.

In the case of the mold created by Witowski JS et al. [20], the total cost was estimated to be USD 150. The article published by Pacioni A et al. [19] did not specify the cost of the materials for making the mold, only the cost of the soft silicone liver phantom at USD 100. The mold made by our team is much more complex (in terms of being far closer to the clinical reality), easy to use, and can be used for casting a very large number of phantoms using multiple substances with a cost of about EUR 270 (cost of materials).

The cost of making a gelatin-based liver phantom with tumors and vessels inside is EUR 8–15. If a large number of liver models are produced (i.e., for a workshop), the price can decrease by up to 20–25%. The price varies exclusively based on the purchase price of the raw materials. In the case of bi-component silicone liver models, the price is much higher, being between EUR 65 (if 10 liver phantoms are made) and EUR 85 (if only one liver phantom is made).

The gelatin-based phantoms can be stored for several weeks in the refrigerator without significant degeneration according to Bude and Alder [21]. We confirmed that the gelatin liver phantoms we made can be properly used for training after being stored for more than 4 weeks in a refrigerator.

Unlike the mold made by Witowski JS et al. [20] for a patient’s preoperative planning, our mold is designed to cast a very large number of soft liver phantoms for training. The cost difference between our mold and the one created by Witowski JS et al. [20] is due to their different dimensions. Ours is larger; each piece has dimensions of 30/10/10 cm (L/W/H) and a thickness of 5 mm. The other has parts with a maximum size of <20 cm and a maximum thickness of 3 mm. The mold components made by our team have been designed with a thickness of 5 mm for greater strength over time and with extensions for easier and faster assembly and disassembly, features that give it high resistance over time for very numerous castings. Fixing and sealing the four parts of our mold is very simple and effective, with screws and sanitary silicone.

The mold made by Witowski et al. [20], unlike the one made by our team, used a cyanoacrylate-based adhesive along with adhesive tape, and it was single-use.

Our mold can be used more than 100 times (tested) without any damage. Our mold is also printed to the highest fidelity with a high-precision professional printer (with a layer thickness of 0.256 mm and a deviation <1%) and a large square printing base of 380 mm (StratasysFortus 380mc). The volume of the liver in our case is 1534 cm^3^, unlike the one made by Witowski, J.S. et al. [20], with a volume of 1289 cm^3^. Thus, we compared the three molds, their realization, and the cost of the liver molds made with them (Table 2).

The gelatin-based liver phantoms created using the modular and reusable 3D-printed mold designed by our team can be used for the acquisition/improvement of ultrasound procedures skills in doctors’ training, such as for diagnostic US and US-guided procedures on the liver. Additionally, the liver phantoms can be used for training in other image-guided procedures. With this mold, we can also create liver phantoms from materials other than gelatin, e.g., silicone.

For the training of surgeons in new procedures, there are high-quality phantoms (very expensive) [5,6,7,8] and phantoms that can be further enhanced in terms of imaging quality and blood flow functionalities, as shown in [34,35,36,37]. The most common 3DP transparent patient-specific liver phantoms are made for pre-surgical planning and usually at a lower scale because of the high costs and long printing time [38,39]. There is a model developed for radiological tissue mimicking [40] and artificial substances for ultrasound properties mimicking, such as attenuation and scattering effect [41]. Also, there are 3DP phantoms for organs other than the liver available for training, such as hand soft tissue for peripheral venous catheter in children [42]. The phantoms created with the 3DP mold developed by our team provide a very accurate model for early-stage training environments. They are simple, easy to cast, easy to use, and cost-efficient. These phantoms are preferred for the education of young surgeons/doctors.

Living animal models (e.g., pigs) have been used for a long time in laparoscopic surgery training [43], as well as in US and imaging training [44,45]. In some experiments, liver tissue has been used for different simulated procedures [46,47] and new technologies [43]. There is no phantom better than a living organ. At the same time, there is no way to create specific conditions on a living pig liver, like multiple tumors of various sizes for imaging-guided puncture/biopsy training, dilated bile ducts for US-guided drainage catheter insertion, cysts or abscesses for US drainage, etc. For this reason, a complete course on US or image-guided procedures should have the following three parts: the first should be theoretical, the second should involve training on liver phantoms with various levels of difficulty, and the last part should be on living anesthetized pigs.

## 5. Conclusions

Life-sized liver phantoms, made from various materials and cast in a modular 3DP reusable mold, are a low-cost alternative to expensive phantoms. The gelatin-based model presented in this paper was validated based on the responses of 33 trainees who participated in hands-on courses during the International Surgical Congresses, with overwhelmingly positive feedback. As a result, we are confident that this liver phantom cast in a 3DP mold can positively contribute to the education of young doctors, who can safely learn new procedures, improving the overall outcome of patients with different liver pathologies.

## Figures and Tables

**Table 1:** Imaging assessment of gelatin liver phantoms using several imaging devices compared to the same procedures on real patients.

	Examination/ Procedure	Examination/Procedure on Gelatin Liver Phantom	Examination/Procedure on Real Liver
1	US examination	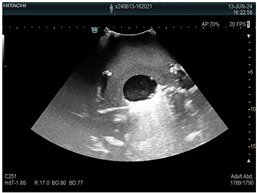 *	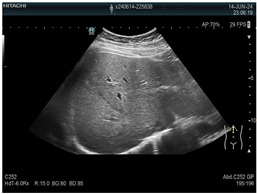 *
2	Elastography	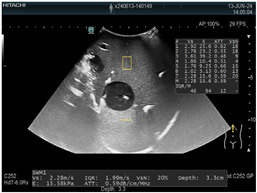 *	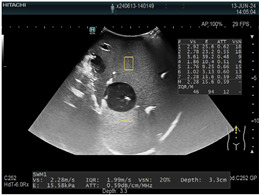 *
3	Fibroscan	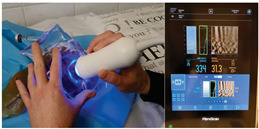 *	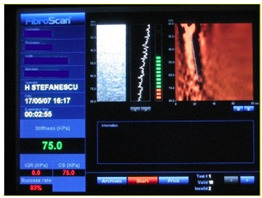 **
4	US-guided tumor puncture/biopsy	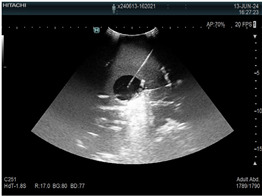 *	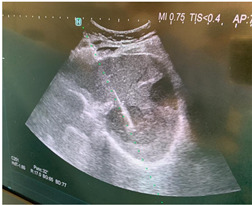 *
5	RFA needle insertion into the tumor	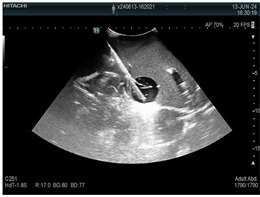 *	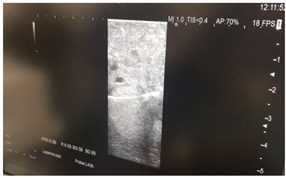 *
6	US-guided Biliary drainage	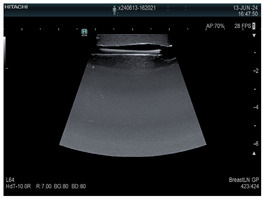 *	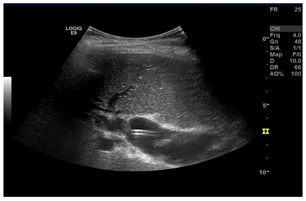 ***
7	Laparoscopic US-guided tumor puncture/biopsy	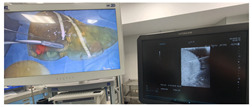 *	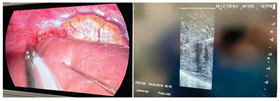 *
8	CT-scan examination	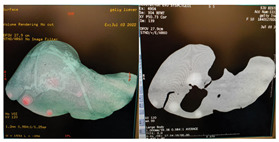 *	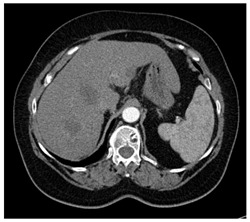 4*
9	MRI examination	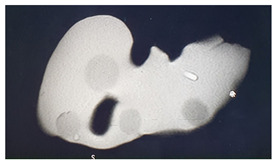 *	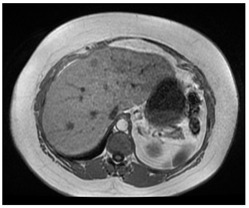 4*
10	US-CT fusion examination	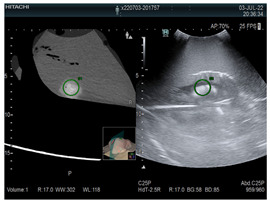 *	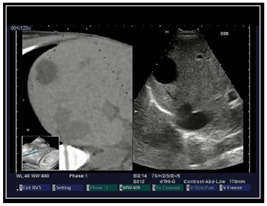 5*
11	CT-guided tumor puncture/biopsy	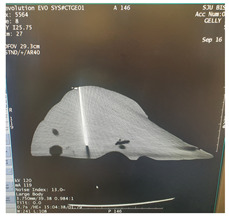 *	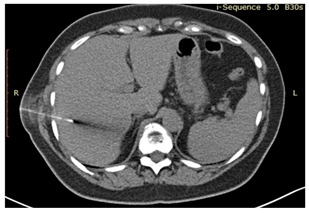 6*

* Personal archive (Dr. Radu Claudiu Elisei). ** Courtesy of Dr. Horia Stefanescu (“Prof. Dr. O. Fodor”, Regional Institute of Gastroenterology and Hepatology, Cluj-Napoca, Romania). *** Courtesy of Dr. Tudor Mocan (“Prof. Dr. O. Fodor”, Regional Institute of Gastroenterology and Hepatology, Cluj-Napoca, Romania). 4* Archive of Bistrita Emergency Clinical County Hospital, Romania. 5* Courtesy of Hiatchi Ltd./Romania. 6* Courtesy of Dr. Andrei Roman (Oncology Institute Cluj-Napoca, Romania).

**Table 2:** Comparison of the three molds.

	This Study	Witowski, J.S. et al. [20]	Pacioni, A. et al. [19]
3D printing technology/material	FDM/PLA (polyactid)	FDM/ABS	FDM/ABS
Layer thickness	0.256 mm (high quality)	Not specified (lower quality)	Not specified (lower quality)
Mold parts thickness	5 mm	3 mm	Not specified
Mold parts size	300/100/100 mm	<200 mm (largest dimension)	Not specified
Total 3D printing time/printing jobs	42 h/2 print jobs	72 h/6 print jobs	Not specified
Single use/multiple use mold	Multiple use	Single use	Multiple use
Liver phantom volume	1534 cm^3^	1289 cm^3^	Not specified
Type of material that can be poured into the mold	Gelatin based, silicon based	Silicon based	Silicon based
Cost of materials for the mold	EUR 270	USD 45	Not specified
Cost of a gelatin phantom	EUR 8–15	Not applicable	Not applicable
Cost of a silicon phantom	EUR 85	USD 150 (mold + phantom)	USD 100

## Data Availability

The data presented in this study are available on request from the corresponding author (graurf@yahoo.com).
